# Construction of Highly Active Interfaces on Screen-Printed Carbon Electrodes via Controllable Electrochemical Exfoliation for High-Performance Flexible Enzyme-Free Glucose Sensing

**DOI:** 10.3390/mi17020251

**Published:** 2026-02-16

**Authors:** Wenjing Xue, Ziyan Chen, Xiao Peng, Haocheng Yin, Yimeng Zhang, Yuming Zhang

**Affiliations:** 1 Key Laboratory of Wide Band-Gap Semiconductor Materials and Devices, School of Microelectronics, Xidian University, Xi’an 710071, China; 25251110156@stu.xidian.edu.cn (W.X.); chenziyan@stu.xidian.edu.cn (Z.C.); zhangyimeng@xidian.edu.cn (Y.Z.); zhangyuming@xidian.edu.cn (Y.Z.); 2 OPPO Guangdong Mobile Communications Co., Ltd., Chang’an Town, Dongguan 523860, China; pengxiao1@oppo.com; 3 Guangzhou Institute of Technology, Xidian University, Guangzhou 510555, China

**Keywords:** electrochemical roughening, screen-printed carbon electrodes, flexible electrode, enzyme-free glucose detection, gold nanoparticles

## Abstract

Enzyme-free flexible glucose sensors hold great promise in the field of wearable health monitoring. However, their performance is limited by the balance between the catalytic interface activity and stability. This paper reports a strategy for interface gradient roughening of screen-printed carbon electrodes (SPCE) via controllable electrochemical exfoliation (EE). It systematically reveals the inherent relationships among the degree of EE treatment, electrode morphology, surface chemistry, and electrochemical performance. On this basis, the deposition of gold nanoparticles (AuNPs) with high density and uniform distribution is achieved, and a high-performance flexible enzyme-free glucose sensor is constructed. The study finds that EE treatment can significantly increase the true surface area of the electrode and introduce abundant oxygen-containing functional groups, thus effectively reducing the charge transfer resistance. Nevertheless, excessive exfoliation leads to the degradation of the conductive network, indicating the existence of a critical “performance window”. The EE-SPCE optimized with 150 cycles has both a high active area and good electrical conductivity, providing an ideal deposition substrate for AuNPs, increasing their distribution density by approximately 158% and reducing the average particle size to 125 nm. The fabricated AuNPs/EE-SPCE sensor exhibits excellent performance in glucose detection: it has a high sensitivity of 550.766 μA·mM^−1^·cm^−2^ in the range of 0.1–3 mM, a detection limit of 0.0998 mM, a wide linear range, excellent selectivity, long-term stability, and good mechanical flexibility. This research not only develops an efficient and scalable method for constructing flexible sensing interfaces but also clarifies the trade-off relationship among “roughening–conductivity–catalytic performance” at the mechanistic level, providing an important theoretical basis and a general strategy for rationally designing high-performance flexible electrochemical devices.

## 1. Introduction

Enzyme-free glucose sensors have received extensive attention in modern medicine, health monitoring, and food industry applications due to their operational stability superior to traditional enzyme-based systems [1,2,3,4]. Although enzyme sensors have high selectivity, the sensitivity of enzymes themselves to environmental conditions, limited service life, and technical challenges in the immobilization process restrict their application in long-term dynamic monitoring [5,6,7]. However, existing research on enzyme-free glucose detection mainly focuses on rigid electrode systems (such as glassy carbon electrodes or metal wires) [8,9,10]. Most sensors on flexible substrates still rely on the enzymatic mechanism [11,12,13]. Since enzyme-free flexible electrodes often struggle to maintain efficient and stable catalytic activity and structural integrity under mechanical deformation [14], developing truly robust flexible enzyme-free sensors still poses challenges.

The key to achieving high-performance enzyme-free flexible glucose sensing simultaneously lies in the rational design of the catalytic interface. Noble metal nanomaterials, especially AuNPs, are regarded as ideal catalysts due to their excellent electrocatalytic activity and anti-poisoning ability [15,16,17]. SPCE shows broad prospects in the field of electrochemical sensing, especially in point-of-care testing and wearable devices, because of their low cost, easy mass production, and good flexibility characteristics [18,19,20]. However, SPCEs are mainly prepared using laboratory-made graphite-based carbon pastes or commercial carbon inks. For either type of slurry, differences in their specific components and subsequent printing and curing processes will significantly affect the electrochemical performance of the electrodes. The inherently chemically inert surface and relatively smooth morphology of SPCEs severely limit the effective deposition of AuNPs, resulting in uneven distribution and low loading, ultimately restricting the further improvement of sensor performance [21]. Pretreating SPCE is a necessary operation to maintain excellent surface performance of the electrode. Currently, pretreatment methods for SPCE, such as strong acid–strong base oxidation or plasma treatment, often have limitations like complex processes, poor controllability, or high equipment requirements. Moreover, most of them focus on surface chemical modification [22,23,24]. However, how to simultaneously achieve the coordinated regulation of the physical morphology and surface chemistry of SPCE through a simple and controllable method, and thus jointly enhance the surface performance of carbon electrodes and the deposition efficiency of AuNPs in the field of enzyme-free modification, remains a key scientific problem to be solved urgently. Electrochemical exfoliation, as a common and effective surface roughening strategy, can significantly increase the effective surface area of electrode materials [25,26]. Nevertheless, this method usually focuses on the roughening of metal surfaces, involves complex procedures or uses non-screen-printed porous carbon layers, and has problems such as poor adhesion and limited scalability [26,27,28]. More critically, there is currently a lack of fundamental understanding of how the degree of electrochemical roughening systematically affects the interface properties of SPCE. How to balance the competitive effects between the increase in surface area and charge transfer efficiency within different treatment ranges in this process has become a core scientific challenge.

In response to the above challenges, this paper proposes a simple and controllable electrochemical roughening strategy. We combine EE with SPCE and achieve precise customization of the flexible SPCE interface by regulating the number of cyclic voltammetry scanning cycles. This study goes beyond simply demonstrating performance improvement and systematically reveals the inherent relationships among the degree of EE, interface properties, and sensing performance. Innovatively, through a single electrochemical process, an ideal substrate interface with both a three-dimensional porous structure and abundant oxygen-containing functional groups is simultaneously constructed, and an optimal treatment window is established, providing a novel solution to the key problem of low deposition efficiency of AuNPs on SPCE. We believe that by precisely regulating the degree of EE in this study, an optimal SPCE interface can be constructed. This interface strikes a balance between an increased specific surface area and maintaining good charge conduction ability, thus laying the foundation for achieving efficient and uniform deposition of AuNPs and ultimately obtaining a high-performance flexible enzyme-free glucose sensor.

## 2. Materials and Methods

### 2.1. Electrode Fabrication and Materials

**Figure 1:**
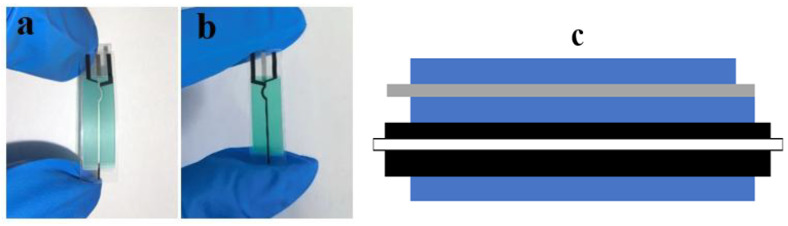
Schematic diagrams of a single flexible SPCE electrode. (**a**) Front view of the electrode; (**b**) Back view of the electrode; (**c**) Schematic cross-sectional structure of the electrode. The white part represents the substrate, the black part represents the carbon electrode, the gray part represents the Ag/AgCl reference electrode, and the blue part represents the insulating film.

The flexible SPCE used in this study was fabricated by Changsha Sanjun Electronics Technology Co., Ltd. (Changsha, China). A colorless transparent polyimide film (thickness: 125 μm) served as the flexible substrate. The working electrode (WE) and counter electrode (CE) were printed using a carbon ink paste composed of conductive carbon black, graphite powder, a polymer binder, and organic solvents. The pseudo-reference electrode (RE) was fabricated from an Ag/AgCl paste containing silver powder (1–3 μm), AgCl particles, and glass frit (as a sintering aid). A PET film (thickness: 10 μm) was laminated as the insulating layer, simplifying the manufacturing process compared to printed insulators. The electrode design features a “sandwich structure” with the WE and RE/CE printed on opposite sides of the substrate (Figure 1), a configuration that minimizes signal crosstalk compared to conventional side-by-side SPCE layouts. The dimensions of the RE, CE, and WE were 0.3 mm × 3 mm, 0.4 mm × 3 mm, and 0.4 mm × 3 mm, with corresponding thicknesses of 3 μm, 10 μm, and 8 μm, respectively.

### 2.2. Physical and Chemical Characterization

The surface morphology of the electrodes and the deposited AuNPs was characterized using a Helios G4 CX focused ion beam scanning electron microscope (FIB-SEM) (Thermo Fisher Scientific Brno s.r.o., Brno, Czech Republic) and a field-emission scanning electron microscope (FE-SEM) (Thermo Fisher Scientific Brno s.r.o., Brno, Czech Republic). SEM imaging was performed with an accelerating voltage of 5.0 kV, a beam current of 43 pA, and a working distance of 4.1 mm. The surface chemistry and elemental states of the electrodes after EE treatment were analyzed by X-ray photoelectron spectroscopy (XPS) (ULVAC-PHI, Inc., Chiba, Japan) and Fourier-transform infrared spectroscopy (FTIR) (Bruker, Ettlingen, Germany).

### 2.3. Electrochemical Measurements and Procedures

All chemicals, including HAuCl_4_·3H_2_O, ethanol (99.5%), KCl (99.9%), K_3_[Fe(CN)_6_] (99.8%), K_4_[Fe(CN)_6_] (99.8%), H_2_SO_4_ (0.5 M standard solution), NaOH (95%), and D-(+)-glucose (C_6_H_12_O_6_, 99.5%), were purchased from MilliporeSigma (Merck KGaA, Darmstadt, Germany). Ascorbic acid (AA, 99.99%), uric acid (UA, 99.0%), and acetaminophen (APAP, 99.0%) were obtained from Shandong Keyuan Biochemical Co., Ltd. (Shandong Keyuan Biochemical Co., Ltd., Heze, China).

All electrochemical measurements were conducted using a Gamry Reference 620E potentiostat (Gamry Instruments, Inc., Warminster, PA, USA) with a conventional three-electrode system, where the SPCE served as the WE, with its integrated carbon and Ag/AgCl electrodes acting as the counter and reference electrodes, respectively. Before modification or testing, the electrodes were sequentially rinsed with deionized water, ethanol, and deionized water again, followed by drying under a gentle nitrogen stream.

The basic electrochemical characterization of the electrodes was performed using cyclic voltammetry (CV) in two different solutions: 1 M KCl (as an inert electrolyte solution, IES) and 1 M KCl containing 5 mM K_4_[Fe(CN)_6_] and 5 mM K_3_[Fe(CN)_6_] (as a redox couple solution, RCS). Voltage range: −0.2–0.6 V, sweep rate: 100 mV/s. The EE treatment was carried out by performing CV scans in 0.5 M H_2_SO_4_ within a potential window of −0.3 V to +1.4 V (vs. the integrated Ag/AgCl RE) at a scan rate of 200 mV/s for a specified number of cycles (10, 50, 100, 150, 200). For the modification with AuNPs, the WE was immersed in a 1 mM HAuCl_4_ solution (in 0.5 M H_2_SO_4_), and a constant potential of +1.0 V was applied for 90 s. The dynamic detection of glucose was performed using chronoamperometry (CA) at an applied potential of +0.3 V in 0.1 M NaOH under continuous stirring. All data were processed and plotted using Gamry Echem Analyst 2, OriginPro2024, and Microsoft Excel software.

## 3. Results and Discussion

### 3.1. Gradient Regulation of Electrode Interfaces by Electrochemical Exfoliation Method

#### 3.1.1. Evolution of Surface Morphology and Structure of SPCE

Electrochemical exfoliation, as an effective interface engineering strategy, enables the systematic regulation of the surface properties of SPCE. To quantitatively analyze the impact of the degree of EE on the electrode interface, we systematically characterized SPCEs subjected to EE treatment with different numbers of cycles (0, 10, 50, 100, 150, 200 cycles).

**Figure 2:**
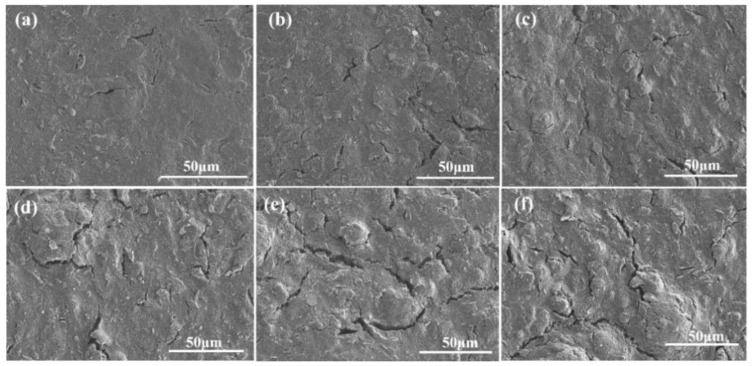
SEM images of the WE after different numbers of EE cycles. (**a**) 0 cycles; (**b**) 10 cycles; (**c**) 50 cycles; (**d**) 100 cycles; (**e**) 150 cycles; (**f**) 200 cycles.

Before conducting electrochemical performance tests, we first systematically observed the surface morphology of the WEs of SPCEs treated with different numbers of EE cycles through scanning electron microscopy. As shown in Figure 2, the surface of the untreated original electrode presented a relatively dense and flat morphology, with only the microscopic texture inherent to the screen-printing process visible (a). After 10 cycles of CV treatment, local etching marks began to appear on the surface, forming a shallow-layer pore structure (b). When the number of treatment cycles increased to 50, significant changes occurred on the electrode surface, with obvious layered exfoliation and a honeycomb-like porous structure emerging (c). As the number of cycles further increased to 100–150, a fundamental transformation occurred in the surface morphology, resulting in a well-developed three-dimensional reticular pore structure and abundant nanoscale trenches (d, e). It is worth noting that when the number of treatment cycles reached 150, a well-developed and stable three-dimensional reticular structure had formed. When the number of cycles was further increased to 200, no further significant changes were observed in the surface morphology (f), indicating that the roughening process had reached a stable stage. This gradual evolution of the surface morphology with the increase in the number of treatment cycles directly confirms the effective roughening effect of the EE process on the surface of SPCE.

#### 3.1.2. Evolution of Electrochemical Performance

**Figure 3:**
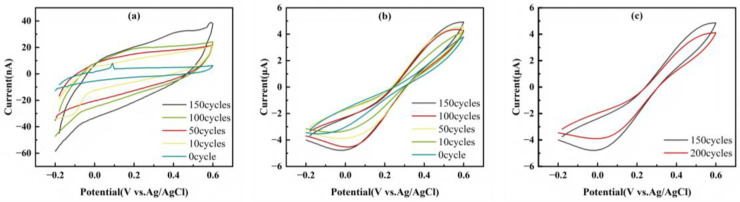
(**a**) CV diagrams in IES after 0–150 cycles of EE; (**b**) CV diagrams in RCS after 0–150 cycles of EE; (**c**) CV diagrams in RCS after 150 and 200 cycles of EE.

To systematically investigate the impact of electrochemical roughening on the performance of SPCE, we first characterized the treated electrodes using cyclic voltammetry in two typical electrolytes to explore their interfacial double-layer characteristics and Faradaic reaction behaviors, respectively. In the inert electrolyte (1 M KCl), the current response of the cyclic voltammogram mainly stems from the double-layer charging process at the electrode/solution interface. As shown in Figure 3a, with the increase in the number of EE cycles, the closed area of the CV curve increased significantly. A quantitative analysis of the charging current (Table 1) revealed that its value increased sharply from 2.920 µA at 0 cycles to 21.165 µA at 200 cycles, an increase of nearly 7-fold. The double-layer charging current is directly proportional to the true electrochemical active area of the electrode, and this order-of-magnitude increase is direct evidence of the successful roughening of the electrode surface. In the solution containing the [Fe(CN)_6_]^3−^/^4−^ redox probe, the peak current on the CV curve corresponds to the Faradaic process. As shown in Table 1, as the number of treatment cycles increased from 0 to 150, the Faradaic current continuously increased from 3.604 µA to 5.744 µA, an increase of approximately 59% (Figure 3b). This indicates that the roughened SPCE exhibits a significantly enhanced response sensitivity to electroactive species in the solution. This phenomenon suggests that the large specific surface area formed by roughening provides more opportunities for the probe molecules to come into contact with and react, promoting the interfacial electron transfer rate and enhancing the intrinsic catalytic activity of the electrode. However, Figure 3c shows that when the number of cycles continued to increase to 200, the signal current decreased. Combining with the SEM results, excessive exfoliation may lead to the merging of cracks to form isolated carbon platforms, causing the effective electrochemically active area to decline due to the damage of the conductive network. Although both the Faradaic current and the charging current increase with the increase in the number of treatment cycles, the growth rate of the background (charging) current is higher, resulting in a decrease in the signal-to-noise ratio from 1234.247 at 0 cycles to 281.367 at 150 cycles. The decrease in the signal-to-noise ratio reveals a fundamental change in the interface properties of the electrode: from the original smooth and low-activity surface to a three-dimensional heterogeneous surface with a high specific surface area and high activity. For the pretreatment process aimed at providing a substrate for the subsequent deposition of AuNPs, a large specific surface area and abundant active sites are far more important than the high signal-to-noise ratio of the initial bare electrode. This three-dimensional structure will create ideal conditions for the uniform and high-density deposition of gold, and the final sensor performance will be dominated by the properties of the gold/carbon composite interface.

**Figure 4:**
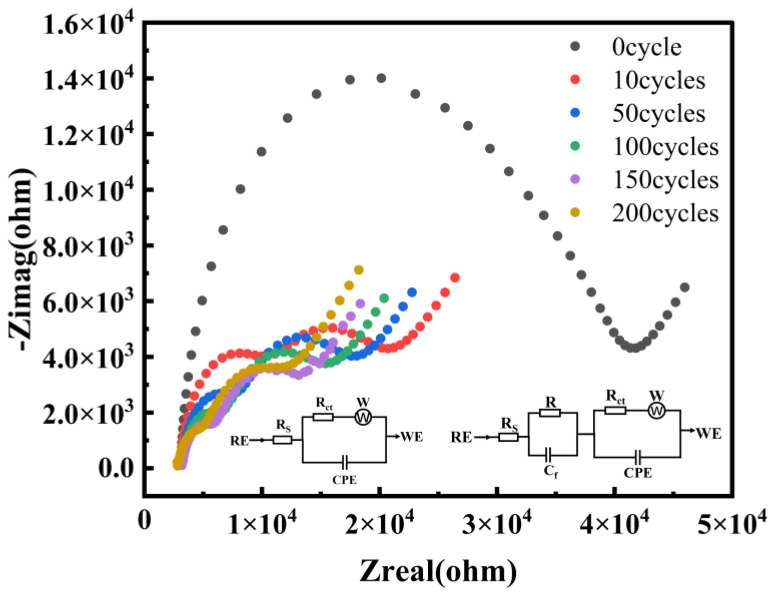
Nyquist plots in the RCS solution after treatment with different numbers of cycles. The inset shows the equivalent circuit of the EIS data.

Based on the cyclic voltammetry analysis, we further conducted a more in-depth quantitative study of the electrode/solution interface using electrochemical impedance spectroscopy (EIS). The obtained EIS data were fitted using the Randles equivalent circuit (inset in Figure 4). The left inset shows the fitting circuit for the electrode surface without EE treatment, and the right inset shows the fitting circuits for the electrode surfaces after 10, 50, 100, 150, and 200 cycles of treatment respectively. The key parameters are summarized in Table 2. The Nyquist plot shown in Figure 4 indicates that the untreated original SPCE presents a semicircle with a large diameter, indicating a relatively high interfacial electron transfer resistance. As the number of CV treatment cycles increases, the diameter of the semicircle in the high-frequency region decreases significantly, reflecting a remarkable improvement in the interface properties. Through quantitative analysis, it was found that the charge transfer resistance decreased sharply from 34.380 kΩ of the original electrode to 3.316 kΩ after 200 cycles, a decrease of 90.4%. This result is completely consistent with the conclusion of the significant increase in the Faradaic current in the aforementioned CV, jointly confirming that the roughening treatment has greatly improved the electron transfer kinetics of the electrode. At the same time, the double-layer capacitance, which characterizes the electrochemical active area, also changes significantly. The fitting data show that the Cdl value increased from 150.5 nF to 4.688 μF after 200 cycles, an increase of approximately 30-fold. This result is highly consistent with the sharp increase in the charging current measured in 1 M KCl, jointly verifying the substantial increase in the true surface area of the electrode from different measurement perspectives.

#### 3.1.3. Analysis of Surface Chemical State

**Figure 5:**
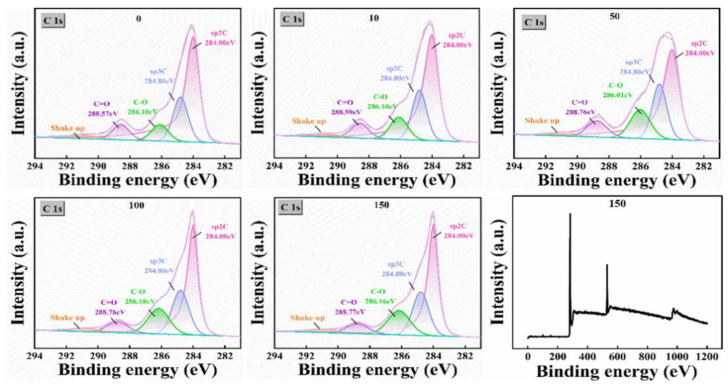
XPS fitting results of the electrodes after different numbers of EE cycles.

Furthermore, we utilized XPS to analyze the changes in surface elements and functional groups of the electrodes with the variation in EE parameters. The results are shown in Figure 5 and Table 3. As the number of cycles increased to 150, the content of sp^2^-C, which represents the ordered graphite structure, decreased from 61.95% to 49.73%, while the sp^3^-C representing the defective structure and oxygen-containing functional groups continued to increase. Among them, the C-O functional group significantly increased from 8.17% to 17.34%, becoming the dominant surface species. This change in surface chemical state is consistent with the electrochemical test results. On one hand, the extensive introduction of sp^3^-C defects and oxygen-containing functional groups such as C-O provides the electrode with abundant electrochemical active sites. This directly explains the internal reasons for the 90.4% reduction in charge transfer resistance and the 59% enhancement of Faradaic current. On the other hand, the oxidative etching of the carbon skeleton and the modification of functional groups jointly promote the formation of nano-scale pore and trench structures. This results in an approximately 30-fold increase in the double-layer capacitance and a substantial increase in the effective specific surface area. This three-dimensional rough interface with both a high-activity surface area and abundant oxygen-containing functional groups not only significantly improves the electron transfer kinetics and interface wettability of the electrode, but also provides ideal anchoring sites for the uniform nucleation and high-density deposition of AuNPs. It effectively fixes the Au(III) precursor through coordination, thus laying a solid foundation for constructing a high-performance enzyme-free glucose sensing interface.

### 3.2. Stage-Wise Evolution of Interface Characteristics and Determination of the Optimal EE Window for SPCE

The aforementioned SEM, CV, EIS, and XPS results have revealed the profound impact of the EE process on the SPCE interface from different perspectives. In this section, a comprehensive discussion of these results will be carried out. The aim is to uncover the underlying evolution mechanism and establish the optimal EE treatment window for the subsequent deposition of AuNPs.

**Figure 6:**
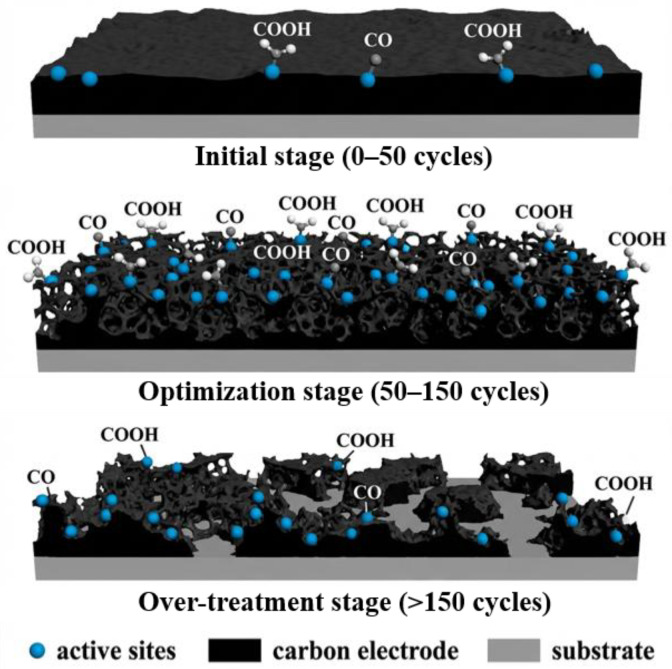
Process of interface evolution.

As shown in Figure 6, based on the systematic characterization data, we propose that the interface evolution of SPCE during the EE process is an evolutionary process involving three typical stages. In the initial stage (0–50 cycles), the electrochemical oxidation at high potential initiates local etching of the carbon skeleton and the preliminary introduction of oxygen-containing functional groups, leading to a sharp decrease in charge transfer resistance and a preliminary expansion of the electrochemical active area. Entering the optimization stage (50–150 cycles), the physical etching and functional group modification have a synergistic effect, constructing a well-developed three-dimensional porous structure and achieving the full activation of the surface chemical state. This enables the electrode to maintain excellent electrical conductivity while obtaining a maximized active area and catalytic site density.

However, when entering the over-treatment stage (>150 cycles), the continuous etching begins to damage the continuity of the carbon skeleton and the conductive network. Some pore walls become thinner or rupture, and isolated carbon islands appear, resulting in structural collapse and performance degradation. Therefore, the treatment condition of 150 cycles is precisely at the end of the optimization stage. It maximizes the interface performance while effectively avoiding the over-treatment risk, establishing an ideal substrate for the subsequent efficient deposition of AuNPs.

**Figure 7:**
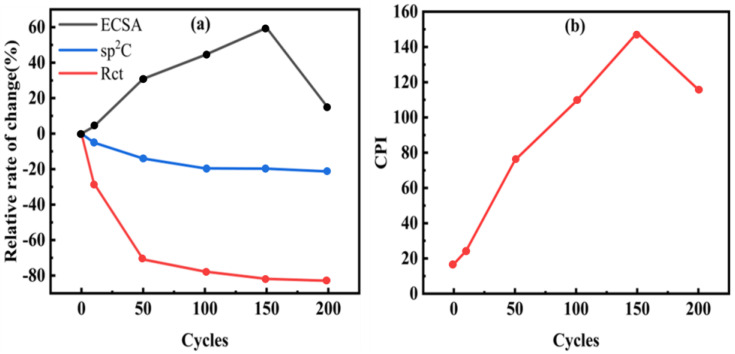
Influence of exfoliation cycles on the interfacial properties and overall performance of SPCE. (**a**) Variation of key parameters with the number of EE cycles; (**b**) Variation of comprehensive performance with the number of EE cycles.

To quantitatively analyze this evolutionary process, we extracted key parameters and plotted their trends with the number of EE cycles (Figure 7a). It can be noticed that the ECSA, representing the surface area, and the Rct, representing the interfacial electron-transfer ability, change most dramatically in the range of 0–50 cycles. The ECSA increases rapidly while the Rct decreases significantly, indicating that this stage is dominated by interface activation. In the range of 50–150 cycles, the growth of ECSA slows down but still maintains a stable upward trend, and the Rct continues to improve to the optimal level. This stage is the synergistic optimization period. After more than 150 cycles, the growth of ECSA tends to saturate, and the structural stability of the electrode begins to be challenged. The local damage to the conductive network leads to a decrease in charge transfer efficiency, signaling the onset of over-treatment.

Nevertheless, a high-performance sensing interface requires the co-existence of a large specific surface area and efficient charge-conduction ability. To find this balance point, we defined a comprehensive performance index (CPI) = ECSA/Rct. This index takes into account the synergistic contributions of the active area and the electron-conduction efficiency simultaneously. The larger the value of this index, the more excellent the comprehensive performance of the electrode. As shown in Figure 7b, the CPI reaches its peak at 150 cycles, clearly identifying the optimal EE window. Beyond this range, although the ECSA still remains at a relatively high level, the negative effects caused by the impaired electrical conductivity will outweigh the benefits brought about by the increase in the surface area, resulting in a decline in the comprehensive performance index. This phenomenon verifies the inevitability of the performance degradation of the electrode in the over-treatment stage.

In this study, the five selected cycle numbers are designed to cover the typical evolution stages from initial activation to over-treatment. The emphasis is on uncovering the overall trends and turning points of the interface performance as the degree of treatment varies. Although no additional gradients were set between 150 and 200 cycles, the above-mentioned data clearly demonstrate that after 150 cycles, the improvement in interface performance approaches saturation, while the structural stability starts to face challenges. This provides sufficient grounds for determining the optimization window.

Based on the above-mentioned analysis, Table 4 summarizes the comprehensive enhancement of the SPCE interface characteristics under the optimal treatment parameter (150 cycles of EE). Therefore, we selected 150 cycles as the optimized parameter for the subsequent gold deposition experiments. This ensures that the electrode can obtain a well-developed three-dimensional porous structure and excellent electrochemical performance, while effectively avoiding the risk of structural damage caused by over-treatment. This treatment method has independently transformed the SPCE into an optimized substrate with high electrical conductivity, a large active area, and abundant surface functional groups. This interface not only has excellent charge transfer capabilities on its own, but more importantly, it provides an ideal physical and chemical environment for the efficient and uniform deposition of subsequent AuNPs and the expression of high activity. It is a prerequisite for constructing a high-performance composite catalytic interface.

### 3.3. Enhanced Deposition and Electrocatalytic Performance of AuNPs on Optimized EE-SPCE

Next, based on the optimized interface created by 150 cycles of EE treatment, we investigated its effectiveness as a substrate for AuNPs deposition in the subsequent electrocatalytic oxidation of glucose.

**Figure 8:**
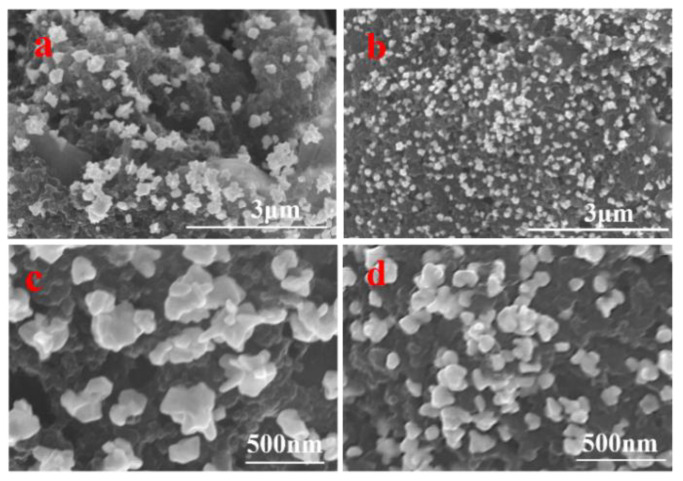
SEM images of the surface after depositing AuNPs. (**a**,**c**) Electrodes without EE treatment; (**b**,**d**) Electrodes treated with 150 cycles of EE.

The SEM images in Figure 8 provide direct visual evidence of the profound impact of EE pretreatment. On the pristine SPCE (Figure 8a,c), the deposited AuNPs exhibited severe agglomeration, forming large, irregular clusters with an average diameter of 312 ± 7.2 nm and a low density of only 65 particles/μm^2^. In sharp contrast, the surface of EE-SPCE (Figure 8b,d) supported uniformly and densely distributed, well-dispersed spherical AuNPs. The average particle size was significantly reduced to 125 ± 3.8 nm, while the density increased sharply to 168 particles/μm^2^, approximately a 158% increase in density compared to the unmodified electrode. This remarkable improvement can be attributed to the synergistic regulatory effect of the three-dimensional rough interface constructed by EE and the abundant oxygen-containing functional groups it introduced. The sharply increased surface area provides more nucleation sites for Au^3+^ ions, while the specifically enhanced anchoring centers (such as C=O, C-O) effectively guide uniform nucleation and inhibit the random migration and coalescence of particles during the electrodeposition process. Ultimately, this optimized nucleation and growth kinetics not only increases the catalyst loading but also, by forming nanostructures with a larger electrochemically active specific surface area and more accessible catalytic sites, lays a crucial structural foundation for enhancing the intrinsic catalytic activity of the electrode. Therefore, the EE treatment not only increases the loading but also achieves a “qualitative” improvement of the catalyst, which is the structural basis for enhancing its intrinsic activity.

**Figure 9:**
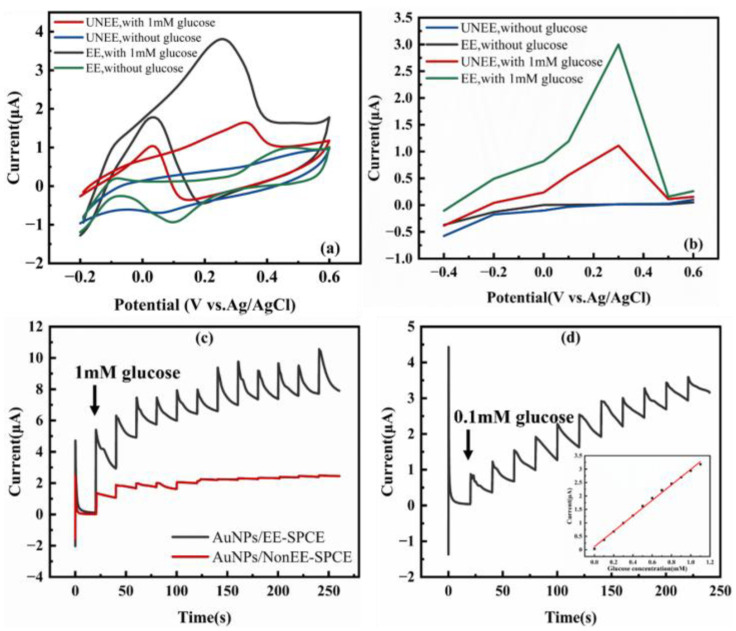
Detection of glucose in 0.1 M NaOH. (**a**) CV results of electrodes with and without EE before and after adding glucose; (**b**) Currents before and after adding glucose at different constant voltages; (**c**) Continuously adding 1 mM glucose at a constant voltage of +0.3 V; (**d**) Continuously adding 0.1 mM glucose at a constant voltage of +0.3 V. The inset shows the calibration curve between current and glucose concentration.

We used 0.1 M NaOH (pH ≈ 13) as the detection medium for the glucose detection experiments. This was based on the fact that under this condition, the electrochemical pathway of gold-catalyzed glucose oxidation is the most well-defined and efficient. It is conducive to clearly quantifying and comparing the intrinsic electrocatalytic performance of different interface structures while eliminating the interference of complex matrices. This enables the excellent morphology of AuNPs on EE-SPCE to be directly translated into enhanced electrocatalytic activity. The CV curves in 0.1 M NaOH (Figure 9a) show that upon the addition of 1 mM glucose, the AuNPs/EE-SPCE electrode generated a sharp and prominent oxidation peak at 0.26 V, with a peak current of 3.808 µA. Meanwhile, the AuNPs/Non-EE electrode exhibited only a weak and broad response, with a peak current of 1.650 µA and a peak potential of 0.33 V. This represents an approximately 130.79% increase in the glucose oxidation peak current, demonstrating the catalytic enhancement brought about by the EE pretreatment. The observed anodic peak corresponds to the direct electro-oxidation of glucose on the surface of AuNPs, a process that involves the dehydrogenation of glucose to gluconolactone.

To further quantify the catalytic performance and determine the optimal detection potential, we conducted steady-state current measurements at different applied potentials (Figure 9b). The AuNPs/EE-SPCE electrode exhibited significantly higher response currents in the potential range from −0.4 V to 0.6 V. The maximum response for glucose oxidation was determined at +0.3 V (vs. Ag/AgCl), where the current output of the AuNPs/EE-SPCE electrode (3.0 µA) was 170% higher than that of the untreated electrode (1.11 µA). Therefore, this potential was selected for all subsequent amperometric detections. The enhanced current response is a direct result of more accessible and well-dispersed catalytic AuNPs sites on the EE-roughened surface, which facilitates more efficient electron transfer and reactant adsorption.

### 3.4. Sensing Performance of AuNPs/EE-SPCE for Enzyme-Free Glucose Detection

The enhanced interfacial and electrocatalytic properties of the AuNPs/EE-SPCE electrode prompted us to conduct a detailed evaluation of its analytical performance for enzyme-free glucose detection. The dynamic detection of glucose was carried out by chronoamperometry in a 0.1 M NaOH solution under continuous magnetic stirring at a constant rotation speed of 150 rpm and an applied potential of +0.3 V. Thus, the dynamic response, sensitivity, and linear range of the sensor were evaluated.

Figure 9c,d show the typical steady-state current–time curves when glucose was gradually added to the continuously stirred 0.1 M NaOH solution. The AuNPs/EE-SPCE sensor exhibited rapid and sensitive response characteristics. After each addition of glucose, it could reach 95% of the steady-state current signal within 3 s. This response speed benefited from the mass-transfer process promoted by the high-density and well-dispersed AuNPs and their efficient electrocatalytic oxidation. In addition, the sensor showed good operational stability under continuous operation conditions. It was continuously stirred in a 0.1 M NaOH background electrolyte and tested at a constant potential of +0.3 V for 6 h. The baseline current showed only negligible drift, less than 0.84% per hour. This indicates that the sensor is suitable for long-term continuous sensing applications.

A key characteristic of a practical sensor is its linear dynamic range. As shown in the calibration curve (inset of Figure 9d), the AuNPs/EE-SPCE sensor exhibited two distinct linear regions, enabling accurate quantification over a wide concentration range. In the low-concentration range of 0.1 to 3 mM, the sensor demonstrated an extremely high sensitivity of 550.766 μA mM^−1^ cm^−2^ (R^2^ = 0.996). This high-sensitivity region is particularly suitable for detecting physiological glucose levels in biological fluids such as sweat, which typically range from 0.1 to 0.6 mM. For the higher concentration range of 3 to 10 mM, the sensor maintained a robust linear response with a sensitivity of 46.4113 μA mM^−1^ cm^−2^ (R^2^ = 0.992), effectively covering the pathologically elevated glucose levels in blood associated with diabetes. In sharp contrast, the performance of the control electrode was much poorer. Its sensitivity was only 252.237 μA mM^−1^ cm^−2^ in the range of 0–2 mM and 26.07 μA mM^−1^ cm^−2^ in the range of 2–5 mM. Moreover, the AuNPs/Non-EE electrode showed significant signal saturation at high concentrations (>5 mM), a limitation that was successfully circumvented through the EE-induced structural optimization. Notably, the increase in the loading of AuNPs on the EE-pretreated electrode does not simply correspond linearly to the improvement in sensor performance, and the catalytic current response per unit area has increased significantly. This indicates that the leap in performance does not solely stem from the increase in the quantity of the catalyst. More crucially, the intrinsic catalytic efficiency of each AuNP has been substantially enhanced. Combining the previous analyses, the core mechanism underlying the remarkable improvement in sensing performance brought about by EE pretreatment can be attributed to the synergistic effect of interface optimization and catalyst optimization. The high-performance carbon interface constructed by EE, on the one hand, creates an ideal substrate for the catalyst by enhancing charge transfer efficiency and providing abundant anchoring sites. On the other hand, it precisely regulates the deposition process of AuNPs, resulting in highly active catalysts with smaller sizes and more uniform dispersion. Therefore, the final performance is the result of the combined action of the “optimized interface” and the “efficient catalyst loading based on this interface” and interface engineering is the fundamental cause driving this synergistic effect.

We conducted an in-depth mechanistic analysis of the bilinear range phenomenon exhibited by the AuNPs/EE-SPCE sensor in glucose detection. This response characteristic typically reflects the transition of the electrocatalytic oxidation process from surface-kinetics control at low concentrations to mass-diffusion control at high concentrations. In the low-concentration range, the observed ultra-high sensitivity indicates that the glucose oxidation reaction is mainly dominated by the intrinsic catalytic activity of the electrode surface. At this stage, glucose molecules in the solution can fully access and occupy the highly active sites on the surface of AuNPs, and the reaction rate is limited by the adsorption of glucose on the catalyst surface and the charge transfer process. The three-dimensional porous structure provided by the roughened SPCE substrate greatly increases the effective loading and dispersion of AuNPs, thus significantly enhancing the density of active sites available for the reaction, which is the key to achieving high sensitivity. When the glucose concentration increases, the significant decrease in sensitivity marks a shift in the rate-determining step of the reaction. At this point, the active sites on the electrode surface tend to be saturated, and the rapid generation and accumulation of reaction products at high concentrations may form a local diffusion layer on its surface. Therefore, the reaction rate is then limited by the mass transfer of reactants to the electrode surface or the rate of product diffusion away from the electrode surface, resulting in a decrease in the slope of the current response with increasing concentration. In contrast, the AuNPs/Non-EE electrode shows obvious signal saturation at concentrations greater than 5 mM, highlighting the crucial role of EE treatment: the three-dimensional porous interface it constructs not only increases the number of active sites but also significantly optimizes the mass-transfer efficiency through its well-developed pore structure, thus effectively expanding the linear range of the sensor to higher concentration regions. Therefore, the observed bilinear range is not a performance defect but an inherent feature of a high-performance, wide-range sensing interface, demonstrating that the AuNPs/EE-SPCE sensor can accurately quantify glucose over a broad concentration range.

Meanwhile, this dual-linear range has clear practical implications for clinical applications. In the ultra-high-sensitivity range of 0.1–3 mM, its performance perfectly meets the concentration requirements of non-invasive monitoring scenarios such as sweat and interstitial fluid (usually 0.1–0.6 mM). In the extended linear range of 3–10 mM, it fully covers a wide range of clinically relevant concentrations from normal blood glucose to significantly high blood glucose. It is particularly worth noting that according to the clinical diagnostic criteria of the World Health Organization (WHO) and major diabetes societies, diabetes can be diagnosed if the fasting blood glucose is ≥7.0 mM or the blood glucose 2 h after a meal is ≥11.1 mM. Therefore, the effective linear detection range of 0–10 mM of this sensor has fully met the need for reliable quantitative monitoring of the core diagnostic threshold ranges of “normal blood glucose,” “prediabetes,” and “diabetes,” which has direct application value for early screening, daily management, and disease assessment. We are also aware of the extremely high blood glucose levels (>10 mM, and even up to 30 mM) that may occur in poorly controlled diabetes. The slowdown in response of the current sensor when the concentration is >10 mM is a common phenomenon of the saturation of surface catalytic sites. This does not undermine its practical value within the core clinical range, and it also points out the direction for future research. That is, in the future, the linear range can be extended to higher concentrations by further optimizing interfacial mass transfer or adopting a dynamic detection mode to fully cover all pathological states.

The limit of detection (LOD) was calculated using the formula LOD = 3σ/S, where σ was determined by measuring the standard deviation of the current response of the blank solution (0.1 M NaOH) (*n* = 10), and S was the slope (sensitivity) of the calibration curve in the low-concentration range. The calculated LOD was 0.0998 mM. This low LOD highlights the ability of our sensor to detect minute changes in glucose concentration. The significantly expanded linear range and extremely high sensitivity of the AuNPs/EE-SPCE sensor are a direct result of the expanded electroactive surface area and the maximized number of highly efficient catalytic sites, both of which are the achievements of the EE pretreatment. Table 5 demonstrates the performance advantages of the AuNPs/EE-SPCE compared with other glucose sensors.

### 3.5. Selectivity, Stability and Environmental Tolerance

For a glucose sensor to transition from a laboratory prototype to a practical analytical tool, its performance must be robust against common biological interferents and environmental fluctuations. Therefore, we systematically evaluated the selectivity, operational stability, and environmental tolerance of the AuNPs/EE-SPCE sensor.

**Figure 10:**
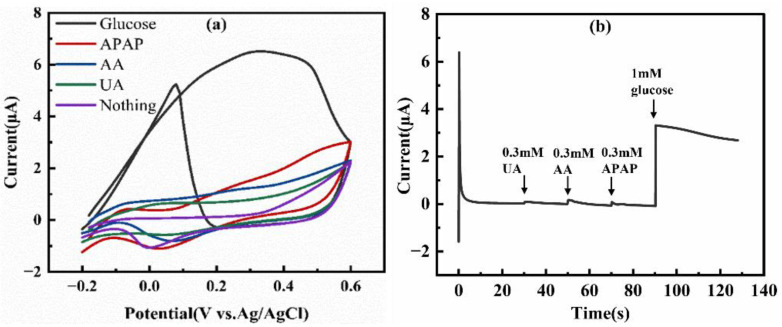
(**a**) CV of AuNPs/EE-SPCE in 0.1 M NaOH containing 1.0 mM glucose, 0.3 mM AA, 0.3 mM APAP, and 0.3 mM UA, respectively; (**b**) Sequential addition of 0.3 mM UA, 0.3 mM AA, 0.3 mM APAP, and 1.0 mM glucose in 0.1 M NaOH.

To evaluate the application potential of the sensor in complex biological fluids such as blood or sweat, this study first conducted selectivity tests against key electroactive interferents. We selected three common substances with high concentrations and strong electrochemical activity in human sweat and blood–uric acid (UA), ascorbic acid (AA), and acetaminophen (APAP). These substances are regarded as the primary and core challenges for evaluating the selectivity of glucose sensors. Their test concentrations were all 0.3 mM, significantly exceeding typical physiological levels. As shown in Figure 10, in the CV profiles, the AuNPs/EE-SPCE electrode did not generate distinguishable Faradaic currents for high-concentration UA, AA, or APAP within the operating potential window, while it showed a clear oxidation peak for 1.0 mM glucose (Figure 10a). The chronoamperometric quantitative analysis at +0.3 V further confirmed this property (Figure 10b). Sequential injection of high-concentration interferents only caused negligible current changes (ΔI < 0.3 μA), while the subsequent addition of 1.0 mM glucose triggered a significant and immediate current response. The excellent selectivity of this sensor stems from the synergistic effect of multiple factors. First, in a strongly alkaline medium, AA, UA, and APAP have high oxidation overpotentials, making their oxidation thermodynamically difficult to occur at +0.3 V [32]. Second, AuNPs themselves have an intrinsic catalytic selectivity for the glucose dehydrogenation process. Third, the abundant oxygen-containing functional groups introduced by the EE process dissociate under alkaline conditions, making the electrode surface negatively charged. This can effectively reduce the non-specific adsorption of similarly negatively charged AA^−^ and UA^−^ ions on the surface through electrostatic repulsion. The above results indicate that this sensor has excellent selectivity towards the most critical electroactive interferents in the physiological environment. It should be noted that the electro-oxidation activities of other common sugars (such as fructose, sucrose, etc.) on gold-based catalysts are usually much lower than that of glucose, and the above-mentioned mechanisms imply a good general resistance to a variety of negatively charged interferents. Nevertheless, there are still numerous other metabolites in complex real-world biological samples. Therefore, future work still needs to conduct more extensive and systematic screening of interferents under conditions closer to real-world applications to ultimately ensure its reliability in complex matrices.

**Figure 11:**
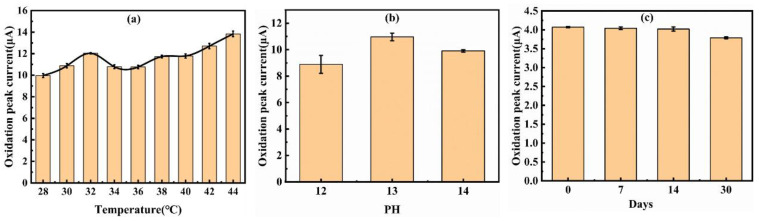
Variations in the oxidation peak current of AuNPs/EE-SPCE in a sodium hydroxide solution containing 2 mM (**a**,**b**) or 1mM (**c**) glucose under different conditions; (**a**) Under different temperatures; (**b**) Under different pH values; (**c**) After being stored for different numbers of days.

Secondly, we studied the detection stability and shelf-life of the EE-optimized electrode. Long-term stability is a significant advantage of enzyme-free sensors. The AuNPs/EE-SPCE electrode demonstrated excellent operational stability. After 30 days of storage under ambient conditions, it retained 93.02% of its initial current response to 1 mM glucose (Figure 11c), and the oxidation peak potential remained consistently at 0.26 V, showing extremely high stability. The minimal performance degradation confirms the robust adhesion of AuNPs to the exfoliated carbon substrate and the inherent stability of the inorganic catalytic interface, effectively circumventing the denaturation problem inherent in enzyme-based sensors.

Thirdly, we investigated the performance of the EE-optimized electrode under different environmental conditions. Figure 11a shows that the oxidation peak current of the electrode for glucose detection remains basically stable in the temperature range of 28–40 °C, fully covering the human epidermal temperature range, and reaches its maximum value at 32 °C, which is exactly the skin surface temperature of human limbs. This indicates that the electrode has great advantages when applied in the field of non-invasive wearable detection. Figure 11b demonstrates the influence of solution pH on the oxidation peak current of the electrode. The oxidation peak current remains at its maximum value at pH = 13. This trend is consistent with the known mechanism of glucose oxidation on gold-based catalysts, in which OH^−^ serves as a necessary reactant in the process of glucose catalysis by AuNPs. As the pH increases, the concentration of OH^−^ increases, which naturally promotes the reaction to proceed to the right and increases the current. When pH > 13, the extremely high alkalinity may lead to the formation of Au oxides or change the reaction pathway of glucose. The complex interaction between reaction kinetics and the surface state of gold results in a decline in electrode performance. In summary, the sensor exhibits optimal performance at pH 13 (0.1 M NaOH), which is consistent with the reported mechanism of gold-catalyzed glucose oxidation in the literature.

Finally, for an enzyme-free glucose sensor intended for wearable applications, it is of crucial importance to maintain the stability of its catalytic and sensing functions under mechanical deformation. To evaluate this performance, we fixed the AuNPs/EE-SPCE sensor on a customized cyclic bending test platform and systematically investigated the impact of bending deformation on its glucose detection performance.

**Figure 12:**
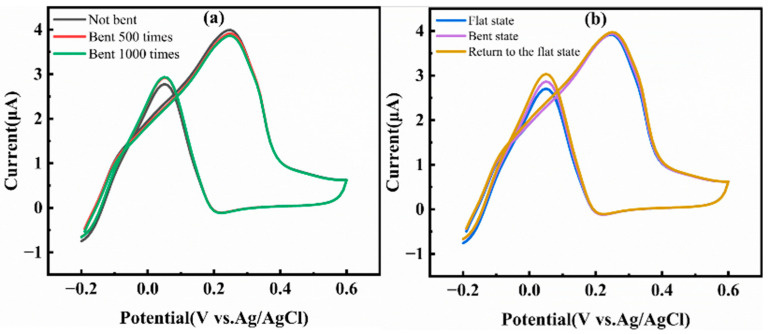
(**a**) CV after different numbers of bends; (**b**) the CV curves of the electrode under different bending states.

We adopted an intermittent testing method, repeatedly bending the sensor with a curvature radius of 5 mm. After each predetermined number of bending cycles, the sensor was restored to a flat state, and its voltammetric response current to 1 mM glucose in a 0.1 M NaOH solution was measured. As shown in Figure 12a, even after up to 1000 bending cycles, the current response retention rate of the sensor remained above 96%. This result indicates that the roughened interface constructed by the EE process and the AuNPs catalytic layer loaded on it possess excellent mechanical robustness. They can withstand repeated bending stresses without detaching from the flexible substrate or deactivating, thus ensuring the long-term stability of the catalytic sites for the glucose oxidation reaction.

To further simulate scenarios that the sensor may encounter during dynamic wearing, we conducted a real-time response test. The sensor was cyclically operated between bending and flat states in a solution containing 1 mM glucose under a potential range of −0.2 to 0.6 V. As shown in Figure 12b, whether in the flat state, the bent state (with a curvature radius of 5 mm), or when restored to the flat state, the oxidation currents generated by the sensor were highly consistent, with a fluctuation range of less than ±2%. This demonstrates that the sensor not only does not experience performance degradation after bending but can even perform the glucose detection task accurately in real-time while in a bent shape.

In summary, the AuNPs/EE-SPCE sensor exhibits excellent properties suitable for practical applications. Its high selectivity significantly reduces false-positive signals that might be caused by key interferents. The outstanding stability ensures the long-term reliable operation of the sensor. Moreover, the robust performance under varying temperature and pH conditions further demonstrates the robustness of this engineered sensing interface. The interface engineering strategy proposed in this study provides an efficient and controllable new method for constructing high-performance flexible enzyme-free sensing interfaces, and reveals the balance relationship among “roughening–conductivity–performance”. Although achieving efficient detection under physiological pH conditions still awaits further breakthroughs, this work, based on the enzyme-free mechanism and flexible electrode materials, has successfully obtained a sensing interface with high activity, high mechanical stability, and good adaptability to the wearing environment. It still highlights an important material and lays a theoretical foundation for the development of practical wearable sensors.

## 4. Conclusions

In this study, we successfully developed an innovative method for the gradient roughening of SPCE via controllable EE. The structure–activity relationship and mechanism of action were systematically elucidated. Through the novel approach of regulating the number of cyclic voltammetry scans in 0.5 M H_2_SO_4_, we achieved a synergistic regulation of the SPCE’s surface morphology and chemical state. Electrochemical oxidation and gas exfoliation worked in tandem to construct a rough, three-dimensional porous surface, boosting the electrode’s effective surface area by nearly 50-fold. Simultaneously, oxygen-containing functional groups predominantly composed of C-O were precisely introduced onto the carbon skeleton, reducing the charge transfer resistance by over 90.4%. For the first time, our research uncovered the stage-by-stage evolution law of electrochemical roughening and determined 150 cycles as the optimal treatment parameter. This parameter maximized the electrode’s active surface area while preventing performance degradation due to over-treatment. The optimized interface, characterized by its high surface area and suitable surface chemistry, proved to be an ideal substrate for AuNPs electrodeposition. It enabled the formation of a dense, uniform, and highly active catalytic layer, directly leading to a sharp increase in the electrocatalytic current for glucose oxidation.

As a result, the final AuNPs/EE-SPCE sensor demonstrated excellent analytical performance, featuring high sensitivity across a wide linear range, a low detection limit, outstanding selectivity, and remarkable long-term stability. EE thus provides an innovative solution to the critical problems of low AuNPs deposition efficiency and poor dispersibility on traditional SPCEs, laying a solid foundation for the fabrication of high-performance enzyme-free glucose sensors. Beyond presenting a high-performance glucose sensor, our findings offer universal guiding principles for the rational design of carbon-based electrochemical interfaces. The elucidated “roughening–conductivity–performance” relationship underscores that in surface roughening treatments, “more is not always better”; instead, optimal performance arises from a delicate balance. This insight, combined with our low-cost and scalable preparation scheme, paves a reliable way for the development of robust flexible sensors, significantly contributing to the progress of wearable health-monitoring technologies.

## Figures and Tables

**Table 1:** CV current changes in electrodes before and after EE under different parameters.

Cycles	IP (μA)	σ_background_ (nA)	SNR	ESCA (mm^2^)
0	3.604	2.920	1234.247	0.327
10	3.747	8.665	432.429	0.340
50	4.707	14.500	324.620	0.428
100	5.193	16.575	313.303	0.472
150	5.744	20.415	281.367	0.522
200	4.121	21.165	194.850	0.374

**Table 2:** Interface parameter values on the electrode surface after treatment with different numbers of cycles.

Cycles	C_f_ (F)	R_s_ (kΩ)	R (kΩ)	Rct (kΩ)	Y_0_	N	W (S∗s^(1/2)^)	Χ^2^
0	0	3.039	0	34.380	133.2 × 10^−9^	0.901	91.07 × 10^−6^	1.07 × 10^−3^
10	150.5 × 10^−9^	3.008	4.992	14.000	3.842 × 10^−6^	0.691	101.9 × 10^−6^	217.8 × 10^−6^
50	2.994 × 10^−6^	3.108	6.958	5.647	355.1 × 10^−9^	0.867	94.10 × 10^−6^	306.2 × 10^−6^
100	3.499 × 10^−6^	3.154	6.088	4.324	495.0 × 10^−9^	0.842	97.19 × 10^−6^	284.2 × 10^−6^
150	4.088 × 10^−6^	3.156	4.989	3.526	658.3 × 10^−9^	0.817	99.35 × 10^−6^	322.6 × 10^−6^
200	4.688 × 10^−6^	2.789	4.392	3.316	1.422 × 10^−6^	0.753	90.55 × 10^−6^	270.9 × 10^−6^

**Table 3:** Detection comparison table of each typical combination in XPS.

Functional Group	0	10	50	100	150
sp^2^ carbon	61.95	58.83	53.25	49.83	49.73
sp^3^ carbon	19.73	20.35	21.32	23.20	22.76
C-O	8.17	10.97	15.32	16.75	17.34
C=O	7.64	7.41	8.70	7.24	7.52
π-π*	2.5	2.44	1.41	2.99	3.15

π-π* represents the shake-up satellite due to π→π* transition in aromatic structures.

**Table 4:** Summary of interface properties and the proposed optimal exfoliation parameters (150 cycles).

Types of Characterization	Key Parameters	Original SPCE	Optimized SPCE	Magnitude of Change	Explanation
Morphological structure	SEM	dense and smooth	A well-developed 3D porous network	N/A	Provides a large anchoring area for AuNPs.
Electrochemical performance	C_dl_	about 0	4.088 μF	effective increase	The electrochemical active area increases sharply.
-	R_ct_	34.380 kΩ	3.526 kΩ	an 89.7% increase	The interfacial electron transfer kinetics is significantly improved.
-	I_P_	3.604 μA	5.744 μA	a 59.4% increase	The number of electrocatalytic active sites increases.
Surface chemistry	Oxygen-containing functional groups	8.17%	17.34%	an 112% increase	Provides a large number of gold nucleation sites
Comprehensive evaluation	Optimal window	not applicable	150 EE cycles	N/A	Strikes a balance between a large specific surface area and good electrical conductivity.

**Table 5:** Comparison of the Glucose Sensor Based on AuNPs/EE-SPCE with Other Sensors.

Modified Electrode	Sensitivity (μA mM^−1^ cm^−2^)	Detection Limit (mM)	Reference
GCE/PANI/GNPs/Cyt c/GOD	63.1	0.01	[29]
AuNPs/GC	87.5	0.05	[17]
AuNPs/GOD–MWCNTs–PVA/GC	16.6	0.2	[30]
Au/3DGFE/ITO	46.6	0.032	[15]
Au/Gold	160	0.5	[31]
AuNPs/SPCE	550.766	0.0998	This work

## Data Availability

The data presented in this study are available on request from the corresponding author.
